# Sleep disturbances and sleep of cognitive decline

**DOI:** 10.1002/alz70860_106227

**Published:** 2025-12-23

**Authors:** Ning Zhang

**Affiliations:** ^1^ Northeastern University, Boston, MA, USA

## Abstract

**Background:**

It remains largely unknown what factors contribute to the speed of progression from mild cognitive impairment (MCI) to Alzheimer's Disease Related Dementias (ADRD), which is known to be highly variable. Growing evidence supports sleep as an important modifiable risk factor for ADRD, and sleep disturbances have been commonly reported in individuals with MCI. The goal of this study was to examine the impact of sleep disturbances on the speed of cognitive decline from the stage of **MCI** to dementia.

**Methods:**

This secondary data analysis is based on the Health Retirement Survey (HRS, 2006‐2022), a nationally representative, biennial survey of Americans aged 50 and older. Sleep disturbances were examined by validated questions in HRS for self‐reported sleep complaints and short sleep duration (<6 hours). The speed of cognitive decline was evaluated by the number of years an individual with MCI remains in the MCI stage before progressing to ADRD. Fewer years indicate faster cognitive decline. MCI was assessed using validated standardized cognitive test scores, and cognitive decline was continuously monitored every two years until reaching dementia. Two‐stage regression analysis was conducted, including: 1) logistic analysis to assess if individuals with sleep disturbances were more likely to have MCI, and 2) survival analysis to test if sleep disturbances in those with MCI contributed to a faster progression from MCI to ADRD.

**Results:**

The existence of sleep complaints and short sleep duration were more prevalent among older adults with MCI, compared to those without MCI. Among individuals with MCI, those with sleep complaints (HR=1.14, 95% CI: 1.03‐1.32) or short sleep duration (HR=1.37, 95% CI: 1.25‐1.51) for at least two waves experienced faster progression from MCI to ADRD, compared to their counterparts with sleep complaints or short sleep duration.

**Conclusions:**

Our data suggest that sleep disturbances in earlier phases of the AD continuum can be valuable indicators of faster cognitive decline, calling for more investigations to understand the role sleep plays in the cognitive decline. Addressing sleep disturbances through targeted interventions may help prevent or delay cognitive impairment for older adults.

